# Strong Association of 677 C>T Substitution in the *MTHFR* Gene with Male Infertility - A Study on an Indian Population and a Meta-Analysis

**DOI:** 10.1371/journal.pone.0022277

**Published:** 2011-07-20

**Authors:** Nishi Gupta, Saraswati Gupta, Madhukar Dama, Archana David, Geeta Khanna, Anil Khanna, Singh Rajender

**Affiliations:** 1 Division of Endocrinology, Central Drug Research Institute (Council of Scientific and Industrial Research), Lucknow, Uttar Pradesh, India; 2 IVF Department, Ajanta Hospitals and IVF Centre Pvt. Ltd., Lucknow, Uttar Pradesh, India; University of Texas School of Public Health, United States of America

## Abstract

**Background:**

Methylenetetrahydrofolate reductase (MTHFR) is an important enzyme of folate and methionine metabolism, making it crucial for DNA synthesis and methylation. The objective of this study was to analyze MTHFR gene 677C>T polymorphism in infertile male individuals from North India, followed by a meta-analysis on our data and published studies.

**Methodology/Principal Findings:**

We undertook genotyping on a total of 837 individuals including well characterized infertile (N = 522) and confirmed fertile (N = 315) individuals. The SNP was typed by direct DNA sequencing. Chi square test was done for statistical analysis. Published studies were searched using appropriate keywords. Source of data collection for meta-analysis included ‘Pubmed’, ‘Ovid’ and ‘Google Scholar’. Those studies analyzing 677C>T polymorphism in male infertility and presenting all relevant data were included in meta-analysis. The genotype data for infertile subjects and fertile controls was extracted from each study. Chi square test was done to obtain odds ratio (OR) and p-value. Meta-analysis was performed using Comprehensive Meta-analysis software (Version 2). The frequency of mutant (T) allele (p = 0.0025) and genotypes (CT+TT) (p = 0.0187) was significantly higher in infertile individuals in comparison to fertile controls in our case-control study. The overall summary estimate (OR) for allele and genotype meta-analysis were 1.304 (p = 0.000), 1.310 (p = 0.000), respectively, establishing significant association of 677C>T polymorphism with male infertility.

**Conclusions/Significance:**

677C>T substitution associated strongly with male infertility in Indian population. Allele and genotype meta-analysis also supported its strong correlation with male infertility, thus establishing it as a risk factor.

## Introduction

Folic acid metabolism is important for stability and integrity of the genome due to its role in maintaining a balance between deoxyribonucleotides (dNTPs) for error free DNA synthesis, DNA methylation pattern and repair [1]. Therefore, deficiency in folate intake or polymorphism(s) in the enzymes of folate pathway may result in aberrant DNA synthesis and methylation. Methylenetetrahydrofolate reductase (MTHFR) is an important enzyme of folate and methionine metabolism, making it crucial for DNA synthesis and methylation. MTHFR reduces methylenetetrahydrofolate to methyltetrahydrofolate which then donates methyl group to homocysteine to form methionine. Methionine is ultimately converted to S-adenosyl methionine which acts as a ‘methyl’ donor for DNA methylation [2]. Also, methylenetetrahydrofolate participates in DNA synthesis by converting uracil to thymine [3]. MTHFR activity is higher in adult testis than other organs in mouse, indicating its critical role in spermatogenesis [2], [4]–[5]. Recent research has identified epigenetic regulation of several genes playing important role in spermatogenesis and fertility [6]. Therefore, aberrations in the *MTHFR* gene could compromise the process of spermatogenesis and predispose the carriers to infertility [7]–[8].

Four SNPs (203G>A, 677C>T, 1286A>C and 1793G>A) in the *MTHFR* gene have been described to affect activity of this enzyme [1], [9]. Of these, 677C>T resulting in the replacement of alanine with valine (A222V, rs1801133), has been studied most often [1]–[4], [9]–[20]. This substitution results in reduced MTHFR specific activity and increased thermolability [21]. Homozygous 677TT variant has ∼30% of residual activity and heterozygous 677CT variant has ∼70% of residual activity when compared to 677CC variant [2], [4], [11], [21]–[22]. This substitution also results in enhanced level of homocysteine and low plasma folate level [10], [20]–[21]. The frequency of 677C>T polymorphism varies with geographical location and the association status with infertility may vary due to ethnic differences [1]–[4], [ 9]–[19]. It has been suggested that low level of folate associated with *MTHFR* polymorphism could be the cause of infertility due to alteration in the synthesis of DNA and RNA molecules [4], [15]. Increase in sperm concentration upon folic acid and zinc sulfate supplementation further suggests the importance of this pathway in spermatogenesis [14]. This is also supported by induction of hypo-methylation by 5-aza deoxy cytidine, which inhibits the differentiation of spermatogonia into spermatocytes in murine model [23].

Most of the studies till date have analyzed this polymorphism in small sample size, giving way to over-estimation of association. We have, therefore, analyzed 677C>T polymorphism in a large sample size (N = 837) to elucidate the correlation between this polymorphic variant and male infertility in Indian population. We also performed a meta-analysis on all eligible published case-control studies, including our data, which established *MTHFR* 677C>T substitution to be a strong risk factor for male infertility.

## Results

### Case-control study

**Figure 1:**
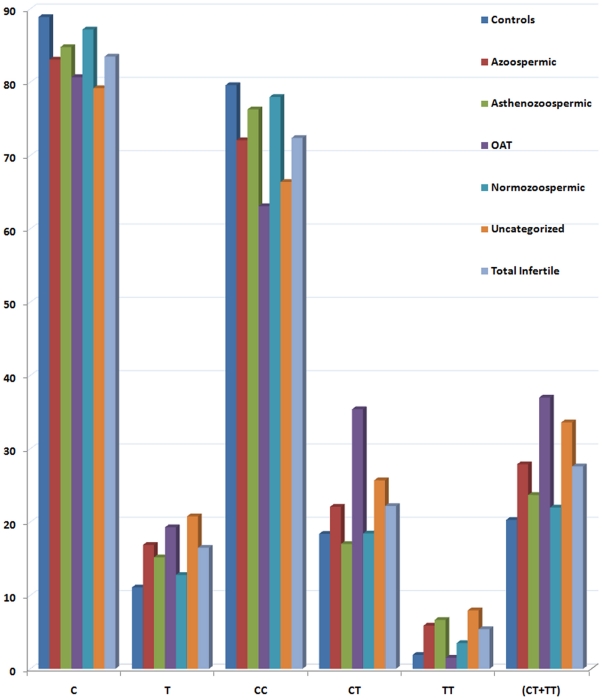
Allele and genotype distribution. Bar diagram showing distribution of allele and genotypes in control and different groups of infertile individuals in our population.

Genotyping by direct sequencing on the DNA samples of all infertile and fertile controls individuals was undertaken. Genotype data for control population fitted well in the Hardy Weinberg Equilibrium equation [p (Exact test)  = 0.246620]. Distribution of mutant allele and genotypes was significantly different between infertile individuals and controls (Figure 1).

Mutant allele (T) was more frequent in infertile men (16.5%) in comparison to fertile controls (11.1%) (Table 1) and the difference was statistically significant (p = 0.0025) with an odds ratio of 1.58 (CI, 1.17–2.13) (Table 1). The frequency of ‘T’ allele in each infertile sub-group was significantly higher in comparison to fertile controls (11.1%) and was almost double (19.3%) in oligoasthenoteratozoospermic (OAT) group, making the difference statistically significant (p = 0.01). Comparison of allele frequency between cases and controls suggested strong association of mutant allele with infertility and with OAT (Table 1).

**Table 1:** Statistical analysis of mutant allele/genotype in our case-control study.

Group (N)	Parameter	C	T	CC	CT	TT	CT+TT
Controls (315)	N (%)	560 (88.9)	70 (11.1)	251 (79.6)	58 (18.4)	6 (1.9)	64 (20.31)
Azoo$ (68)	N (%)	113 (83.1)	23 (16.9)	49 (72.1)	15 (22.1)	4 (5.9)	19 (27.9)
	OR/95% CI		1.63 (0.98–2.72)#		1.33 (0.70–2.53)	3.42 (0.93–12.55)	1.52 (0.84–2.76)
	p-value/Chi-square		0.06/.53		0.39/0.73	0.07/-	0.17/1.91
Astheno (135)	N (%)	229 (84.8)	41 (15.2)	103 (76.3)	23 (17.03)	9 (6.67)	32 (23.7)
	OR/95% CI		1.43 (0.95–2.17)		0.97 (0.57–1.65)	3.66 (1.27–10.53)	1.22 (0.75–1.97)
	p-value/Chi-square		0.09/2.9		0.89/0.02	0.02*/-	0.42/0.65
Normo (141)	N (%)	246 (87.2)	36 (12.8)	110 (78)	26 (18.45)	5 (3.5)	31 (22)
	OR/95% CI		1.17 (0.76–1.80)		1.02 (0.61–1.71)	1.90 (0.57–6.36)	1.11 (0.68–1.79)
	p-value/Chi-square		0.47/0.52		0.92/0.01	0.33/-	0.69/0.16
OAT (65)	N (%)	105 (80.7)	25 (19.3)	41 (63.1)	23 (35.4)	1 (1.5)	24 (37)
	OR/95% CI		1.90 (1.15–3.15)		2.43 (1.35–4.36)	1.02 (0.12–8.70)	2.30 (1.29–4.07)
	p-value/Chi-square		0.01*/6.5		0.002*/9.19	1.00/-	0.00385*/8.35
Uncateg (113)	N (%)	179 (79.2)	47 (20.8)	75 (66.4)	29 (25.7)	9 (7.96)	38 (33.6)
	OR/95% CI		2.10 (1.40–3.15)		1.67 (1.00–2.80)	5.02 (1.73–14.56)	1.99 (1.23–3.20)
	p-value/Chi-square		0.0002*/13.22		0.0485*/3.89	0.003*/-	0.004*/8.12
Total (522)	N (%)	872 (83.5)	172 (16.5)	378 (72.4)	116 (22.2)	28 (5.4)	144(27.6)
	OR/95% CI		1.578 (1.17–2.13)		1.33 (0.93–1.89)	3.10 (1.27–7.60)	1.49 (1.07–2.09)
	p-value/Chi-square		0.0025*/9.14		0.12/2.49	0.0095*/6.73	0.0187*/5.56

# The calculations for mutant allele have been done with reference to allele ‘C’; for mutant genotypes with reference to ‘CC’ between cases and controls.

$ Azoo-azoospermia; Astheno-asthenozoospermia; Normo-normozoospermia; OAT-oligoasthenoteratozoospermia; Uncateg: uncategorized.

*Indicates statistically significant value.

Similarly, the frequency of homozygous mutant genotype (TT) in infertile individuals was almost three times (5.4%) to that of controls (1.9%), and the difference was statistically significant (p = 0.009 at 99% level of confidence). The frequency of heterozygous genotype was also higher in infertile group (22.2%) in comparison to control samples (18.4%); however, the difference was not statistically significant (Table 1). In sub-group analysis, homozygous mutant genotype showed strong association with asthenozoospermia (p = 0.02) and uncategorized infertile (p = 0.003) groups. Overall, the presence of all mutant genotypes (CT+TT) was higher in infertile individuals in comparison to controls, and the difference was statistically significant (p = 0.0187) (Table 1).

### Meta analysis

#### Literature assessment

Twenty nine studies were retrieved by our literature search strategy. Out of these, only 15 had analyzed 677C>T substitution in correlation with male infertility. Two studies were excluded from the analysis as one did not provide detailed information required for meta-analysis [19] and the other was not directly relating the genotypes with infertility [18]. Hence, only 13 studies qualifying our strict selection criteria were included in the analysis. Along with the present study from India, this meta-analysis included data on 3094 cases and 2877 controls. Allele and genotypes data for all these studies were tabulated (Table 2).

**Table 2:** Data extracted from published studies included in the meta-analysis.

Study	Population	Group	Cases						Controls					
			Total	CC	CT	TT	C	T	Total	CC	CT	TT	C	T
Bezold et al, 2001	No Info	Total	255	114	93	48	321	189	200	92	89	19	273	127
Stuppia et al, 2003	Italian	Total	93	37	37	19	111	75	105	33	43	29	109	101
Ebisch et al, 2003	Dutch	Total	77	42	28	7	112	42	113	50	48	15	148	78
Singh et al, 2005	Indian	Total	151	105	40	6	250	52	200	163	37	0	363	37
Park et al, 2005	Korean	Total	373	105	205	63	415	331	396	145	200	51	490	302
Paracchini et al, 2006	Italian	Total	59	11	32	16	54	64	46	18	21	7	57	35
Lee et al, 2006	Korean	Azoo$	174	44	100	30	188	160	325	118	166	41	402	248
		OAT	186	71	81	34	223	149						
		Total	360	115	181	64	411	309						
A et al, 2007	Chinese	Azoo	228	83	97	48	263	193	252	128	95	29	351	153
		Oligo	127	47	63	17	157	97						
		Total	355	130	160	65	420	290						
Dhillon et al, 2007	Indian	OAT	179	81	77	21	259	119	200	70	100	30	240	160
Tetik A et al, 2008	Turkish	Azoo	50	23	22	5	68	32	50	30	20	0	80	20
		Oligo	50	21	22	7	64	36						
		Total	100	44	44	12	132	68						
Ravel et al, 2009	French	Azoo	70	33	31	6	97	43	114	49	52	13	150	78
		Oligo	180	85	70	25	240	120						
		Total	250	118	101	31	337	163						
Safarinejad et al, 2011	Asian	OAT	164	58	80	26	196	132	328	144	148	36	436	220
Gava et al, 2011	Brazilian	Azoo	49	27	15	7	69	29	233	167	53	13	387	79
		Oligo	107	54	45	8	153	61						
		Total	156	81	60	15	222	90						

$ Azoo-azoospermia; OAT-oligoasthenoteratozoospermia; oligo-oligozoospermia.

#### Heterogeneity test and Sensitivity analysis

A true heterogeneity existed between studies for allele (P_heterogeneity_ = 0.00, Q = 39.66, df(Q)  = 13, I^2^ = 67.221, var = 0.001, τ^2^ = 0.050, SE = 0.031,τ = 0.224) and genotype (P_heterogeneity_ = 0.00, Q = 44.44, df(Q)  = 13, I^2^ = 70.75, var = 0.004, τ^2^ = 0.109, SE = 0.064, τ = 0.330) comparisons. The ‘I^2^’ value of more than 50% for between studies comparison in both allele and genotype analysis shows high level of true heterogeneity.

In allele meta-analysis, sensitivity analysis performed by exclusion of the studies involving small sample size [13]–[15], decreased heterogeneity (P_heterogeneity_ = 0.016, I^2^ = 54.07, Q = 21.77, df(Q) = 10, var = 0.000, τ^2^ = 0.025, SE = 0.021, τ = 0.158) to a small extent but exclusion of studies with very high p values [1], [4], [13]–[14] decreased heterogeneity to a large extent (P_heterogeneity_ = 0.136, I^2^ = 33.98, Q = 13.63, df(Q)  = 9, var = 0.000, τ^2^ = 0.012, SE = 0.016, τ = 0.108). Here, τ^2^ defines between studies variance and is used to asses heterogeneity; however, this is not commonly used for this purpose as it depends on the particular effect metric used in the analysis. Var denotes variance and SE denotes standard error for heterogeneity test. Similarly, in genotype analysis exclusion of the studies with small sample size [13]–[15] decreased heterogeneity to a small extent (P_heterogeneity_ = 0.001, I^2^ = 68.07, Q = 31.32, df(Q) = 10, var = 0.003, τ^2^ = 0.082, SE = 0.056, τ = 0.287). As a result of this exclusion, there was no significant difference in the results of fixed effect (OR = 1.361, p = 0.000) and random effect (OR = 1.363, p = 0.004) models. After exclusion of studies with high p values [1], [3], [10], [13]–[14], heterogeneity decreased to a lesser extent (P_heterogeneity_ = 0.002, I^2^ = 67.13, Q = 24.343, df(Q) = 8, var = 0.005, τ^2^ = 0.091, SE = 0.071, τ = 0.301) and the results in random effect model (OR = 1.571, p = 0.000) were almost identical to that of fixed effect model (OR = 1.528, p = 0.000). Sensitivity analysis thus showed that differences in the outcome between the two models (fixed and random effect) could be attributed to a few studies with very small sample size or very high p values. Under the conditions of heterogeneity, random-effects model is more appropriate. However, since there was no change in the inference adopting either model, we present results of both the models. There is no difference in the overall inference except in genotype sub-group analysis for oligozoospermic cases, where we use fixed effect model for drawing inference since random effect model gives more weight to studies with smaller sample size.

#### Meta-analysis using allele frequency

**Figure 2:**
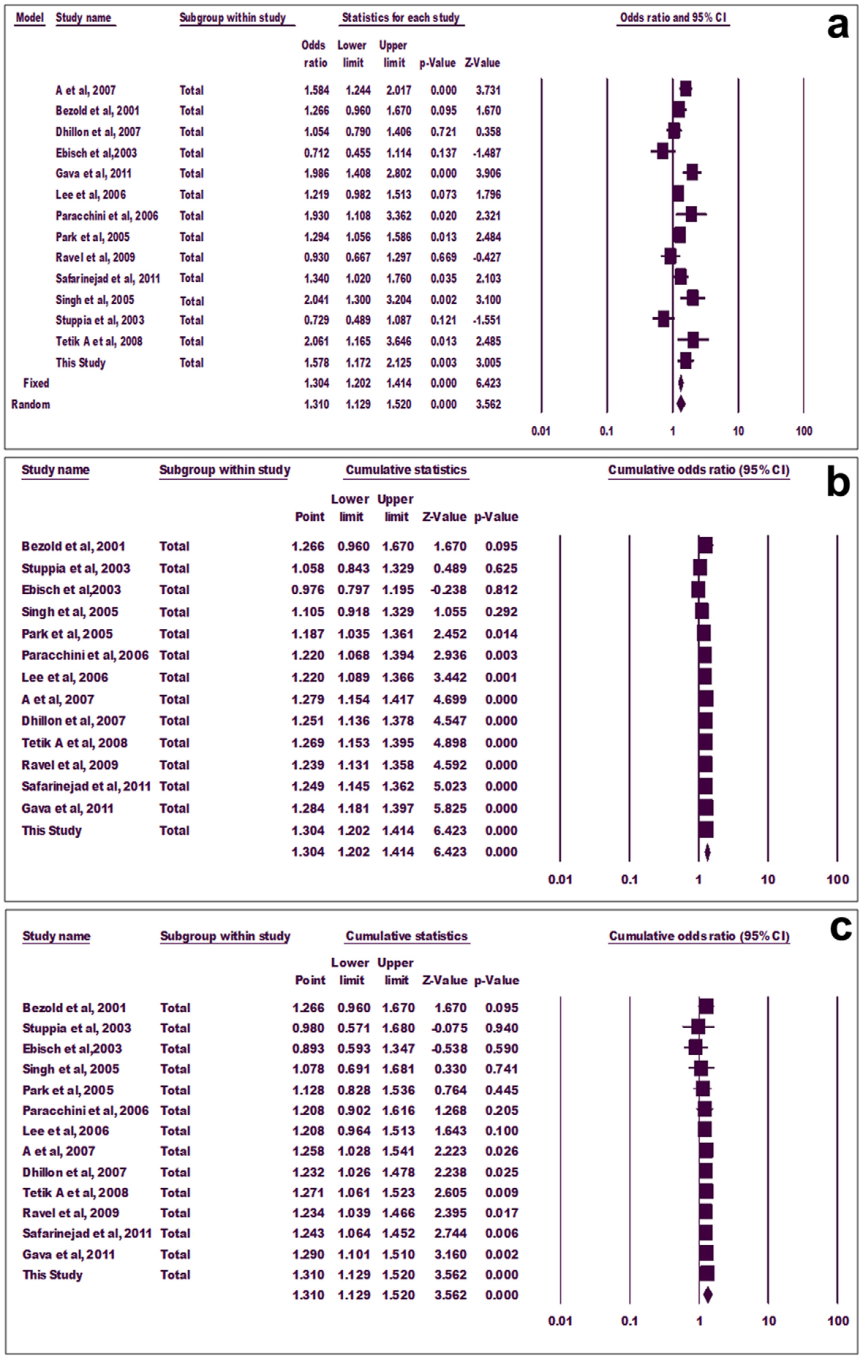
Forest plot. Meta-analysis using allele frequency (a), cumulative allele meta-analysis using fixed effect (b) and random effect models (c).

Mutant allele showed significant association with infertility in both fixed effect (p = 0.000, OR = 1.304, 95% CI = 1.202-1.414) and random effect (p = 0.000, OR = 1.310, 95% CI = 1.129-1.520) models (Figure 2a). In cumulative analysis using fixed and random effect models, the association of mutant ‘T’ allele with infertility turned statistically significant with the addition of study of Park et al (2005) and A et al (2007), respectively, and stayed significant thereafter (Figure 2b and c).

**Figure 3:**
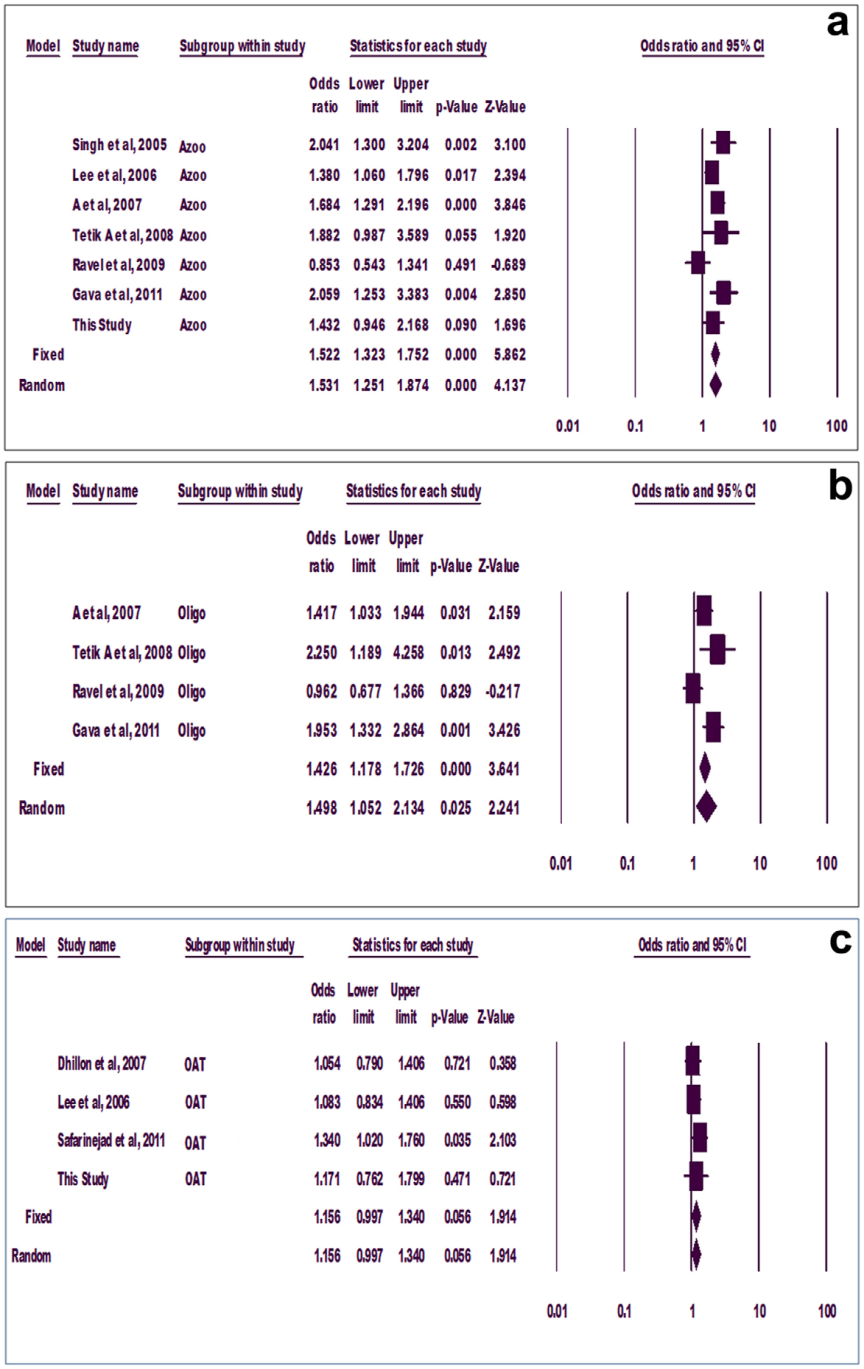
Forest plot. Group-wise allele meta-analysis for azoospermia (a), oligozoospermia (b) and oligoasthenoteratozoospermia (c).

Sub-group analyses were done for azoospermia, oligozoospermia and OAT groups. Few of the previous studies did not categorize infertile subjects into sub-groups; therefore, such studies had to be excluded from sub-group analysis. Significant association of mutant allele with azoospermia was observed using both fixed (p = 0.000, OR = 1.522, 95% CI = 1.32-1.175) and random (p = 0.000, OR = 1.531, 95%CI = 1.25-1.87) effect models (Figure 3a). Similarly, mutant allele showed positive correlation with oligozoospermia adopting both fixed (p = 0.000, OR = 1.426, 95% CI = 1.178-1.726) and random (p = 0.025, OR = 1.498, 95% CI = 1.052-2.134) effect models (Figure 3b). However, mutant allele did not show association with OAT using either model (p = 0.056) (Figure 3c).

#### Meta-analysis using genotype frequency

**Figure 4:**
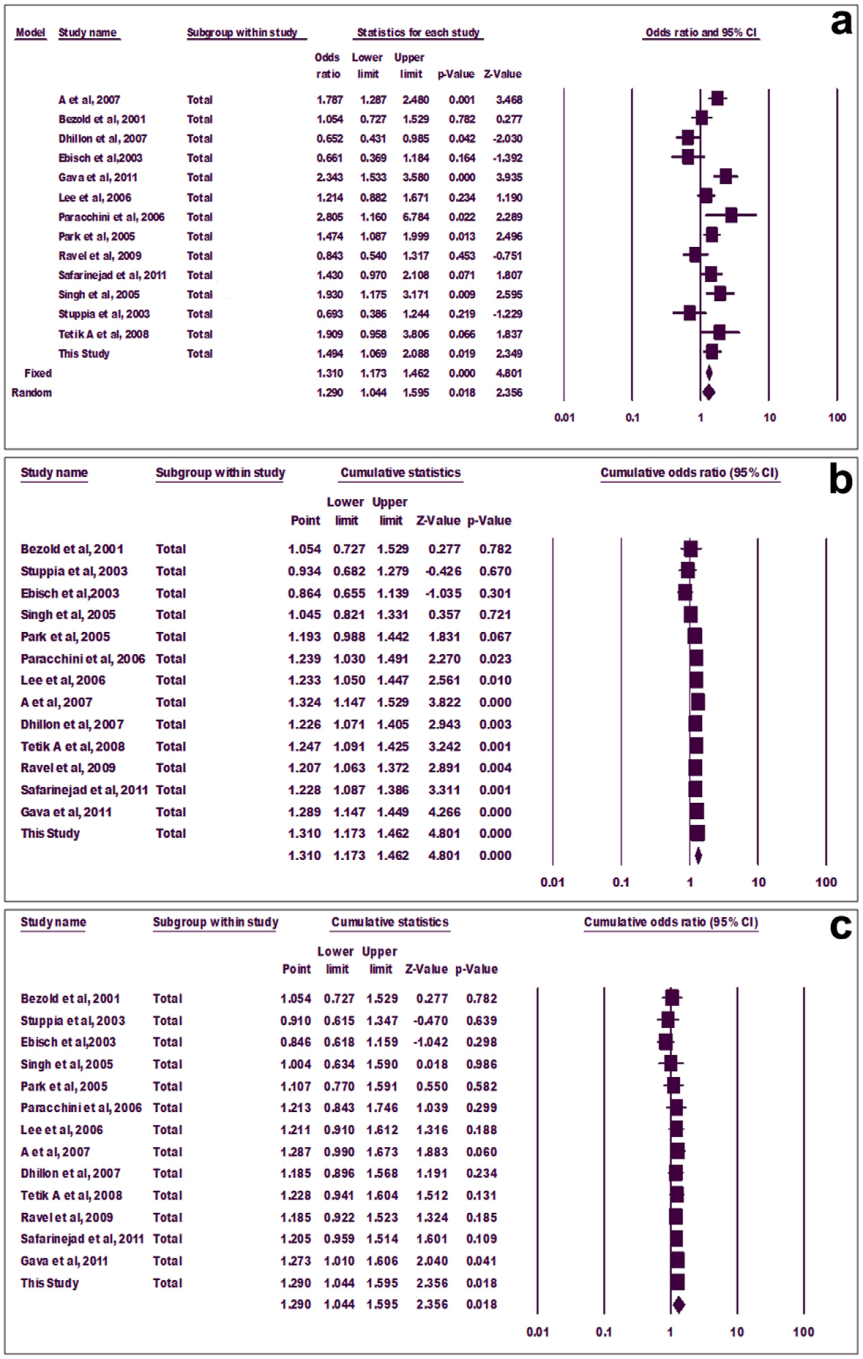
Forest plot. Meta-analysis using genotype frequency (a), cumulative genotype meta-analysis using fixed effect (b) and random effect models (c).

Similar to allele meta-analysis, pooled odds ratio for mutant genotypes (CT+TT) showed statistically significant association with infertility adopting both fixed (p = 0.000, OR = 1.310, 95% CI = 1.173-1.462) and random (p = 0.018, OR = 1.290, 95% CI = 1.044-1.595) effect models (Figure 4a). The fixed effect model cumulative analysis showed that addition of the study of Paracchini et al (2006) turned the overall association significant (p = 0.023) (Figure 4b). With introduction of another two studies, p value became highly significant (p = 0.00, significant at 99% level of confidence) and stayed significant thereafter (Figure 4b). However, with random effect model, the overall association turned significant only after inclusion of the study of Gava et al, 2011 (p = 0.041) and further introduction of our study supported the inference (p = 0.018) (Figure 4c).

**Figure 5:**
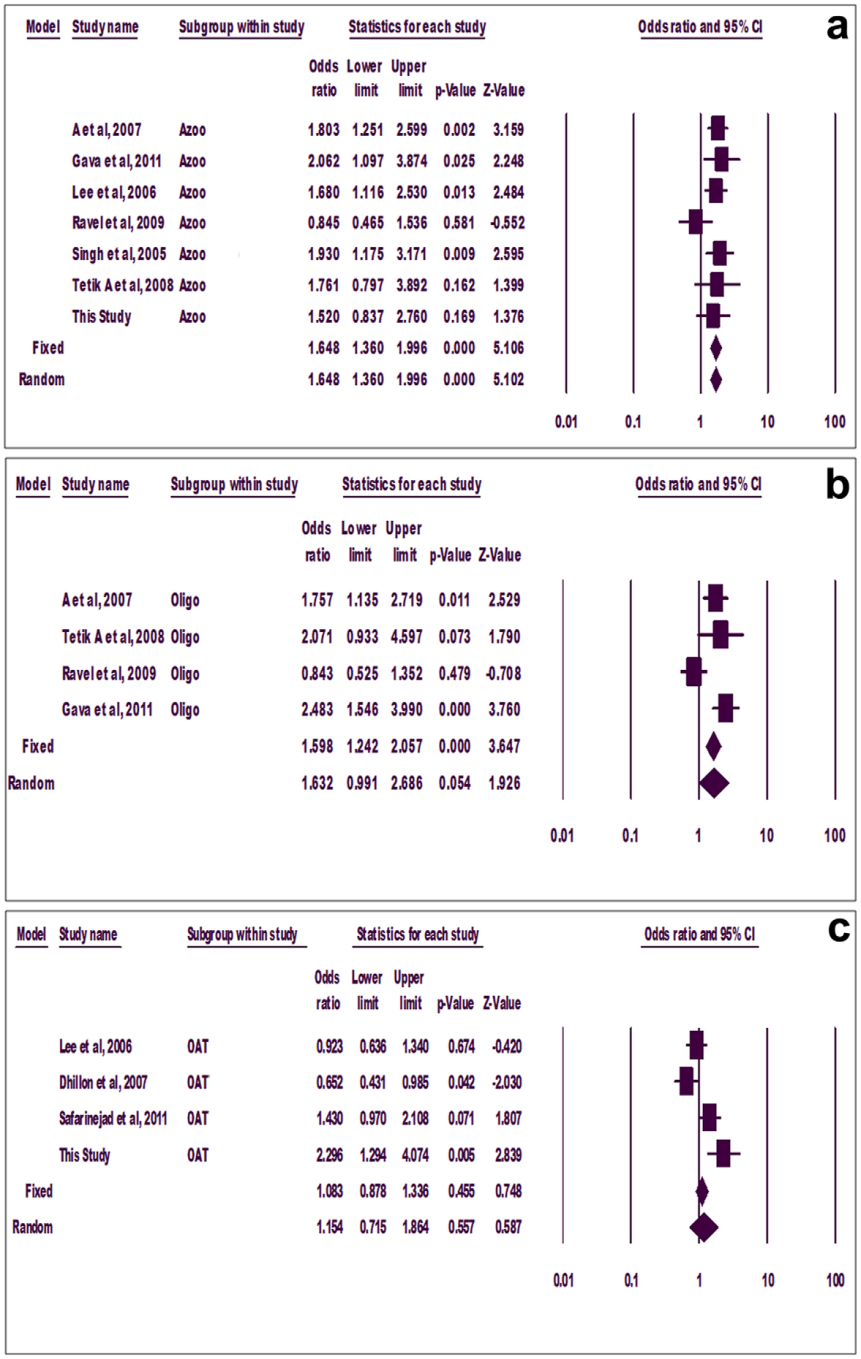
Forest plot. Group-wise genotype meta-analysis for azoospermia (a), oligozoospermia (b) and oligoasthenoteratozoospermia (c).

Sub-group analysis showed significant association of mutant genotypes with azoospermia using both fixed and random effect models (p = 0.000, OR = 1.648, 95% CI = 1.360-1.996) (Figure 5a). Mutant genotypes also associated with oligozoospermia using fixed (p = 0.000) but not random (p = 0.054) effect models (Figure 5b). However, mutant genotypes did not associate with OAT in any case (fixed model, p = 0.455, random model, p = 0.557) (Figure 5c).

#### Publication bias

**Figure 6:**
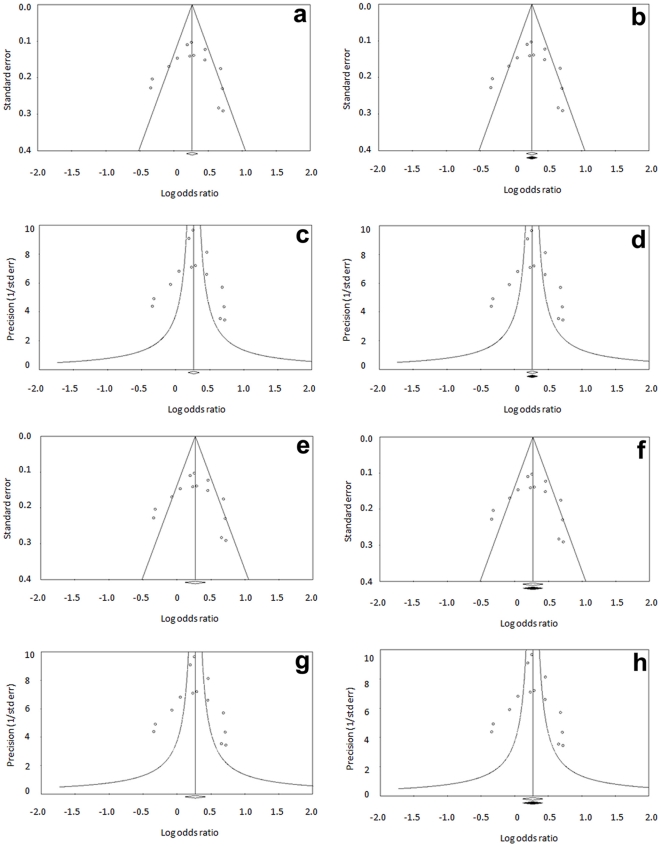
Publication bias (Allele meta-analysis). Funnel plot of standard error by log odds ratio using fixed model for observed set of studies (a) and after imputation (b). Funnel plot of precision by log odds ratio using fixed model for observed set of studies (c) and after imputation (d). Funnel plot of standard error by log odds ratio using random effect model for observed set of studies (e) and after imputation (f). Funnel plot of precision by log odds ratio using random effect model for observed set of studies (g) and after imputation (h).

**Figure 7:**
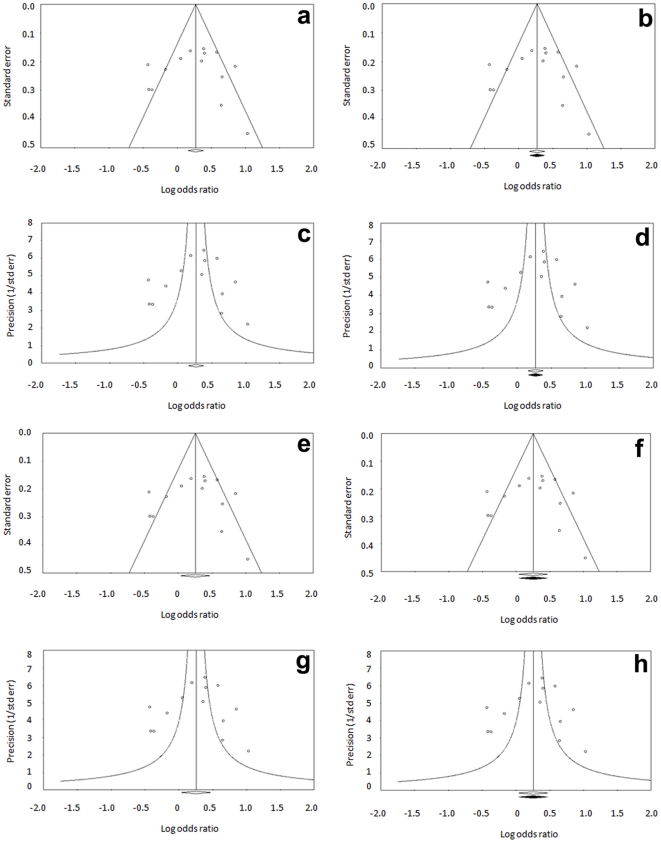
Publication bias (Genotype meta-analysis). Funnel plot of standard error by log odds ratio using fixed model for observed set of studies (a) and after imputation (b). Funnel plot of precision by log odds ratio using fixed model for observed set of studies (c) and after imputation (d). Funnel plot of standard error by log odds ratio using random effect model for observed set of studies (e) and after imputation (f). Funnel plot of precision by log odds ratio using random effect model for observed set of studies (g) and after imputation (h).

We generated funnel plots using standard error and precision values for allele (Figure 6a–h) and genotypes (Figure 7a–h) using both fixed and random effect models. Apart from observed sets of studies, the plots were also drawn after imputation. Symmetrical distribution of studies in the funnel plots suggests absence of publication bias. This is also supported by other tests described below.

In allele analysis, classic fail-safe ‘N’ value of 130 (p = 0.000, Z = 6.28) suggests that 130 null studies would have to be included to nullify the effect or convert the combined ‘p’ to a non-significant (>0.050) value. Orwin's fail-safe ‘N’ to bring observed odds ratio of 1.30 to 1.1 is 25, indicating that at least 25 null studies would be required to bring the effect size to null. Begg and Mazumdar Rank correlation test also showed no evidence of publication bias. Egger's intercept (B0) was 0.422 with t value of 0.253 (one tailed p value = 0.40, two tailed p value = 0.81), further suggesting no publication bias. No change in overall summary estimate (odds ratio) after ‘trim and fill’ procedure suggests absence of publication bias (Figure 6 b,d,f,h).

Similarly, in genotype analysis, no evidence of publication bias was seen. Classic fail safe ‘N’ was 60 (p = 0.00001, z = 4.51), indicating that additional 60 null studies would be required to bring the p value in non-significant range. In the same way, Orwin's fail safe ‘N’ to bring observed odds ratio of 1.31 to 1.1 was 26, indicating that additional 26 null studies would be required to bring effect size to null. Similar to the allele analysis, Begg and Mazumdar Rank correlation test showed no evidence of publication bias. Egger's intercept (B0) was -0.49 with t value of 0.25 (one tailed p = 0.40, two tailed p = 0.80), suggesting no publication bias. No changes in the pooled odds ratio after ‘trim and fill’ procedure (Figure 7 b,d,f,h), confirmed absence of publication bias. Symmetry of the funnel plots and all statistical tests described above showed no trace of publication bias. Therefore, we are confident that there is no over-estimate of the infertility risk associated with this SNP.

## Discussion

Mutant allele and genotypes at 677C>T were strongly associated with male infertility in our population. This was also supported by meta-analysis on previously published studies including our results. The findings of our study become particularly interesting since two previous studies on Indian populations had reported contrasting observations to each other [4], [11]. Singh et al (2005) showed significant association of mutant genotypes with infertility while Dhillon et al (2007) showed no statistically significant difference of 677C>T variants between infertile and fertile males. Dhillon et al, tried to explain the variation in results on the basis that their study included majority of OAT patients in comparison to the majority of azoospermic cases in the study by Singh et al. In the present study, we included both azoospermic and OAT individuals, and had contrasting observations. Our analysis has shown significant association of mutant allele and genotypes with infertility and with OAT in sub-group analysis. Presence of mutant genotypes did not associate with azoospermia in our samples. To the best of our knowledge, the three Indian populations studied so far are not ethnically different. The differences in the results could therefore be attributed to other factors such as variations in recruitment of subjects.

677C>T substitution has been studied in different populations for its possible association with male infertility [1]–[4], [ 9]–[19]. Bezold et al (2001) showed for the first time association of homozygous mutant genotype with male infertility. Several studies later on showed significant association of homozygous mutant genotype with infertility in different populations [2]–[3], [ 11]–[12], [ 15]–[17]. Two studies conducted on Korean population [2], [12] showed significant increase of homozygous mutant genotype in infertile males than fertile controls. Park et al showed that OAT and non obstructive azoospermic patients in unexplained infertile group had higher frequency of homozygous mutant genotype in comparison to explained infertile males. Similarly, a study on Turkish population also inferred homozygous mutant genotype to be a risk factor for infertility characterized by oligozoospermia and non-obstructive azoospermia [16]. Other studies provided evidence for association of this polymorphism with male infertility in the Chinese [2] and Brazilian [17] populations.

In contrast to the above, a study on Dutch population revealed that mutant genotype was not a risk factor for male infertility [14]. Two studies on Italian populations reported contrasting observations [13], [15], one showing no significant association of heterozygous or homozygous mutant genotypes with male sub-fertility [13] and the second reporting increased risk of infertility in individuals with homozygous mutant genotype in comparison to heterozygous and wild type genotypes [15]. Similarly, in a French population, no statistically significant correlation of mutant genotype with male sub-fertility was observed [1]. We found true heterogeneity between studies in both allele and genotype meta-analysis, which is also evident from slight variations in the results of fixed and random effect models. The presence of heterogeneity would force us to use random effect model for further analysis. However, after doing sensitivity analysis we chose to use fixed effect model on the basis that there were few small studies affecting the results of meta-analysis. Since there was no significant difference in the results using either model, we present results of both; however, the inference is based on fixed effect model.

Meta-analysis showed significant association of mutant allele and mutant genotypes (CT+TT) with infertility. Introduction of the studies of Park et al, 2005 and Paracchini et al, 2006 in the allele and genotype cumulative analysis, respectively, provided enough data to conclude that the mutant allele and genotypes are risk factors for infertility. Addition of more studies later on strengthened the conclusion and now this substitution has been established as a risk factor. The strength of our conclusion is also indicated by a very narrow confidence interval for odds ratio. Subgroup analysis has shown significant association of mutant allele and genotypes (CT+TT) with infertility characterized by azoospermia and oligozoospermia but not OAT. Analysis according to geographical distribution of populations could not be undertaken due to relatively lesser number of studies. An earlier meta-analysis on this SNP included eight studies and reported a highly significant association between mutant genotypes and infertility (p = 0.002; OR- 1.23; 95% CI- 1.08-1.41) [24]. A recent meta-analysis on this polymorphism included 10 studies and concluded no overall association between this polymorphism and male infertility [25]. Several observations in the latter study, such as association of this mutation with azoospermia and no association with OAT, are similar to us. However, overall inference in Wu et al., [25] differs from ours as well as from Tuttlemann et al. [24]. Cumulative analysis in our study clearly states less likelihood of finding no overall association as reported by Wu et al. However, inclusion in our meta-analysis of few recent studies and data from our case-control study could be responsible for differences in the overall inference.

It is apparent from our analysis that 677C>T substitution associates strongly with infertility in Indian population and meta-analysis establishes it as a risk factor for male infertility. High fail safe ‘N’ and symmetrical distribution of studies in funnel plots ascertains absence of publication bias, further strengthening our conclusion. The penetration of this SNP could be affected by dietary folate intake and geographical factors such that overall phenotype is an outcome of interaction between these factors. Low dietary intake of folic acid could cause several health problems including but not limited to neural tube defects in developing embryos [26], homocysteine accumulation [27], and impaired DNA synthesis and repair [27]. Low folate level in the Indian and African populations in comparison to the Western and European populations, makes them more susceptible to infertility [11] and other health problems listed above. Inadequate folic acid intake [28] and prevalence of vegetarian diet in India, in coupling with more than 10% overall frequency of this polymorphism, could justify folic acid supplementation for both men and women.

Though exact mechanism by which 677C>T substitution affects fertility is not yet clear, some possible mechanisms have been put forward. Induction of hypo-methylation by 5-aza deoxy cytidine inhibited the differentiation of spermatogonia into spermatocytes in murine model [29], which could explain the association between this SNP and infertility. Since several genes participating in spermatogenesis are regulated by DNA methylation [8], individuals with 677T allele are associated with decreased global genomic methylation [14]. Further, low levels of folate may lead to hyperhomocysteinemia, resulting in oxidative stress. Oxidative stress is well known to cause damage to sperm plasma membrane, and mitochondrial and nuclear DNA [4]. Hyperhomocysteinemia leads to precocious atherosclerosis which results in lower blood flow in testicular arteries, resulting in alteration in spermatogenesis [2]. All the above taken into account partially explains the association between this polymorphism and male infertility. In nutshell, it appears, we have now enough data to conclude that 677 C>T is a risk factor for infertility in general and azoospermia in particular.

## Materials and Methods

### Case-control study

#### Sample Collection

The study was approved by the Institutional Human Ethics Committee of the Ajanta Hospitals and IVF Centre, Lucknow. Before enrolment in the study, each subject's informed written consent was obtained in response to a fully written and verbal explanation of the nature of study. In the case-control study, a total of 837 individuals from north India including 522 infertile and 315 fertile controls of Indo-Aryan ethnicity were recruited. The patients and controls were recruited at the Ajanta Hospitals and IVF Centre Pvt. Ltd. 765, ABC complex, Alambagh, Lucknow. The inclusion criteria of the cases included infertility persisting longer than one year and absence of any obvious fertility problem in the partner. Clinical observations on the female partners including menstruation and ovulation ruled out any problem on female side. Patients having infection of accessory glands, diabetes, hypertension, arthritis, tuberculosis, human immunodeficiency virus, and those on drugs known to influence fertility were excluded. The patients were further categorized in sub-groups as per WHO 1999 criteria [30]. Normozoospermic infertile men (N = 141) had normal semen profile (defined as in the control group except fertility) and infertility of unknown etiology. Asthenozoospermic infertile men (N = 135) had a sperm count >20×10^6^/mL, motility <50%, and > = 30% normal morphology; OAT (N = 65) had a sperm count <20×10^6^/mL, motility <50% and <30% normal morphology; non-obstructive azoospermic infertile men (N = 68) with no sperm in the ejaculate and 113 infertile patients who were not categorized due to lack of one or the other semen parameter. The controls were recruited following the criteria of normal semen profile (WHO 1999) with confirmed paternity.

#### Genomic DNA Isolation and Sequencing

Genomic DNA was extracted from the peripheral blood of patient and control samples using phenol-chloroform-isoamyl method [31]. The point mutation (677C>T) in the *MTHFR* gene was typed using direct DNA sequencing technique. Briefly, primers around the polymorphic site were designed with the help of GENETOOL software. PCR was carried out in a total reaction volume of 10 µl each in thin walled tubes consisting of 1.0 µl of PCR buffer (10X), 1.0 µl of MgCl_2_ (25 mM), 1.0 µl of dNTPs (10 mM), 2.0 pM of each of the forward (5′ CATCCCTATTGGCAGGTTACCC 3′) and reverse (5′ GGGAAGAACTCAGCGAACTCAG 3′) primers, 1.0 unit of *Taq* DNA polymerase enzyme (Applied Biosystems) and 40 ng of genomic DNA. PCR cycling was carried out in ABI Veriti thermal cycler (Applied Biosystems, USA). PCR amplification conditions included denaturation at 95 °C for 10 minutes followed by 35 cycles of denaturation at 95 °C for 30 seconds, annealing at 63°C for 30 seconds and polymerization at 72°C for 40 sec, and a final stage of polymerization at 72°C for 7 minutes. The amplified products were directly sequenced using BigDye™ chain termination chemistry on ABI 3730 DNA analyzer (Applied Biosystems, USA) [32]. Multiple alignment and sequence analysis was done using Auto Assembler Software (Applied Biosystems, USA).

#### Statistical Analysis

Genotype data for control population was analyzed for fitness in the Hardy Weinberg Equilibrium. For this purpose, data was analyzed using calculator available at http://ihg.gsf.de/cgi-bin/hw/hwa1.pl. Chi square analysis was used to compare the allele and genotype data between cases and controls. In addition to the comparison of all the patients with the controls, each sub-group of infertile individuals was compared with controls. Data was analyzed using the Vassar Stats Online Calculator (http://faculty.vassar.edu/lowry/VassarStats.html). P-value of less than 0.05 was considered to be statistically significant.

### Meta-analysis

677C>T has been explored in several studies in different ethnic groups, making it important to conduct meta-analysis. We had used Comprehensive Meta-analysis Version 2 software for this purpose.

#### Identification of studies

A systematic search was done on published literature using the keywords ‘MTHFR and male infertility’, ‘folate metabolism and male infertility’, ‘MTHFR 677C>T polymorphism and male infertility’ through ‘Pubmed’ ‘Ovid’ and ‘Google Scholar’ up to march 2011. Detailed information for each study on 677C>T polymorphism in male infertility such as the purpose and design of the study, presentation of the data, genotyping method used, inclusion and exclusion criteria of the cases and controls was collected. Detailed information, wherever not available, was collected by contacting authors.

#### Inclusion and exclusion criteria

The following inclusion criteria were set for the meta-analysis: (i) each trial is an independent case-control study; (ii) the purpose of all the studies and statistical methods is similar; (iii) it supplied enough information to calculate the odds ratio; (iv) SNP typing was done at high resolution level and (v) inclusion of the patients was done according to the standard diagnosis parameter. The exclusion criteria included: i) study not providing enough information (incomplete raw data) and ii) not well-described.

#### Data Extraction and Statistical Approach

Genotype data for *MTHFR* 677C>T polymorphism related to male infertility was gathered. Information regarding the first author, year of publication, ethnicity of study population, number of cases and controls and allele and genotype frequency was collected.

#### Statistical analysis

Chi-square analysis was performed and the odds ratio with 95% confidence interval was calculated using Vassar Stats online statistical calculators (http://faculty.vassar.edu/lowry/VassarStats.html) for all the possible genotypes. Association analysis was undertaken to compare the frequency of mutant allele and mutant genotypes between cases and controls.

For meta-analysis, major consideration is the type of ‘effect size’ chosen for statistical analysis. In the present study, computed effect size in the form of ‘odds ratio’ and ‘confidence interval’ was chosen. A Chi square based ‘Q’ test defined by Cochran was used to assess the heterogeneity (between study variability) in the meta-analysis. A significance level of P<0.10 instead of traditional P<0.05 was used because of its low power and to avoid type II errors for statistical test of heterogeneity [33]. Since the ‘Q’ statistics is only useful for testing the existence of heterogeneity qualitatively but not quantitatively, another index ‘I^2^’, calculated as the percentage of the total variability in a set of effect sizes due to true heterogeneity, was used to quantify the degree of heterogeneity. A tentative classification of ‘I^2^’ values proposed by Higgins and Thompson has been used to interpret the magnitude; viz. 25%, 50% and 75% which corresponds to low, medium and high heterogeneity, respectively [34]. In the absence of significant heterogeneity determined by the results of Q test, the Mantel-Haenszel fixed effect model (Peto method) was used for the combination of data, while in the presence of significant heterogeneity, the Dersimonian Laird random effect model (DL method) was used for combining the data [34]-[36]. Sensitivity analysis was also done to validate the assumptions and decisions made, and for the robustness of the method used in the analysis.

A comparison of results based on fixed and random effects models before and after exclusion of outlier studies or studies involving small sample size was used as a method for sensitivity analysis [33]. High resolution plots (forest plots) were generated to estimate the pooled odds ratio corresponding to 95% confidence interval and the p value. Both fixed effect and random effect models were used to analyze the data. Cumulative meta-analysis was also done to observe the effect of subsequent addition of each study. Subgroup analysis according to infertility phenotype (azoospermia, oligozoospermia and OAT) was also carried out to estimate the specific odds ratio for a particular sub-group.

Publication bias was investigated by using the funnel plots; viz. funnel plot of standard error by log odds ratio and funnel plot of precision by log odds ratio. Different statistical tests such as Begg and Mazumdar rank correlation, Egger's regression intercept, Duvall and Tweedie's trim and fill procedure and Fail-safe ‘N’ were adopted to assess and quantify the publication bias and its impact on the analysis. The classic fail-Safe N and the Orwin fail-safe ‘N’ assess if the entire observed effect is an artifact of bias. Rank correlation and regression procedures are used for testing the presence of bias. Duvall and Tweedie's trim and fill procedure tests how the effect size will shift, if the apparent bias were to be removed.
